# Disentangling the multigenic and pleiotropic nature of molecular function

**DOI:** 10.1186/1752-0509-9-S6-S3

**Published:** 2015-12-09

**Authors:** Ruth A Stoney, Ryan M Ames, Goran Nenadic, David L Robertson, Jean-Marc Schwartz

**Affiliations:** 1 Computational and Evolutionary Biology, Faculty of Life Sciences, University of Manchester, Manchester, M13 9PT, UK; 2 School of Computer Science, University of Manchester, Manchester, M13 9PT, UK; 3 Wellcome Trust Centre for Biomedical Modelling and Analysis, University of Exeter, RILD Level 3, Exeter, EX2 5DW, UK; 4 Manchester Institute of Biotechnology, The University of Manchester, M1 7DN, UK

**Keywords:** biological function, systems biology, network, pathways, Gene Ontology, pleiotropy, multi-functionality

## Abstract

**Background:**

Biological processes at the molecular level are usually represented by molecular interaction networks. Function is organised and modularity identified based on network topology, however, this approach often fails to account for the dynamic and multifunctional nature of molecular components. For example, a molecule engaging in spatially or temporally independent functions may be inappropriately clustered into a single functional module. To capture biologically meaningful sets of interacting molecules, we use experimentally defined pathways as spatial/temporal units of molecular activity.

**Results:**

We defined functional profiles of *Saccharomyces cerevisiae *based on a minimal set of Gene Ontology terms sufficient to represent each pathway's genes. The Gene Ontology terms were used to annotate 271 pathways, accounting for pathway multi-functionality and gene pleiotropy. Pathways were then arranged into a network, linked by shared functionality. Of the genes in our data set, 44% appeared in multiple pathways performing a diverse set of functions. Linking pathways by overlapping functionality revealed a modular network with energy metabolism forming a sparse centre, surrounded by several denser clusters comprised of regulatory and metabolic pathways. Signalling pathways formed a relatively discrete cluster connected to the centre of the network. Genetic interactions were enriched within the clusters of pathways by a factor of 5.5, confirming the organisation of our pathway network is biologically significant.

**C**onclusions**:**

Our representation of molecular function according to pathway relationships enables analysis of gene/protein activity in the context of specific functional roles, as an alternative to typical molecule-centric graph-based methods. The pathway network demonstrates the cooperation of multiple pathways to perform biological processes and organises pathways into functionally related clusters with interdependent outcomes.

## Introduction

Biological functions must be carried out in a synchronised manner to ensure proper timing of processes like cell division and metabolism. Molecular functions arise from complicated sets of physical interactions between large numbers of proteins, RNAs and various regulatory pathways, which can be difficult to reconstruct, represent and analyse. In systems biology, molecular function is mapped using molecular interaction networks. Protein-protein interaction (PPI) networks are frequently used to map protein functionality [1-5]. Within interaction networks, molecules are usually represented as single nodes connected by physical interactions. Functionally similar nodes tend to cluster together into dense sub-networks, referred to as functional modules [4,6,7] or "pathways" [8], forming the basis of network analysis to study function [3-5]. One aim of identifying sub-networks is to illustrate the position and connectivity that molecules and functional modules have within the network [7]. They are used to examine the organisation of different functions within the cell, showing how information is passed through physical interactions to enable the system to function as a whole. Many studies have used *Saccharomyces cerevisiae *to model functionality [8-11] due to the availability of extensive PPI, genetic interaction (GI) and gene annotation data, making it an ideal organism for developing methods of functional organisation.

A great deal of research has focused on computational methods used to identify clusters/sub-networks based on topological features [12-14]. However, such networks tend to utilise the sum of a molecule's interactions, without accounting for the temporal and spatial nature of its interactions. Simply because two proteins can interact does not mean that they will interact in every context [15]. Clustering approaches tend to treat spatial/temporal edges as if they are constant. These sub-networks, which represent functional modules, may as a result bring together functions that are unrelated in the cell. Evidence for this comes from discrepancies in community detection in networks created from different data types [16]. The combination of different data types has been shown to improve the functional homogeneity of topological clusters.

To deal with the issue of spatial/temporal edges we propose a method using experimentally validated pathways as the units of cellular processes. In this context pathways represent groups of proteins shown to interact under specific experimental conditions. This differs from the definition used in Kelley (2005) [8], in which clusters in PPI networks were described as pathways. In our approach proteins that participate in multiple, context dependent, interactions appear in multiple pathways, rather than being represented by a single highly connected node. Gene Ontology (GO) annotations derived from experimental evidence or sequence homology were used to assign collective functionality to the pathways. Annotated pathways were then connected according to functional overlap. Linking pathways by shared functionality enables us to examine the flow of information among biological functions, giving insight into the organisation of function within the cell.

## Methods

**Figure 1:**
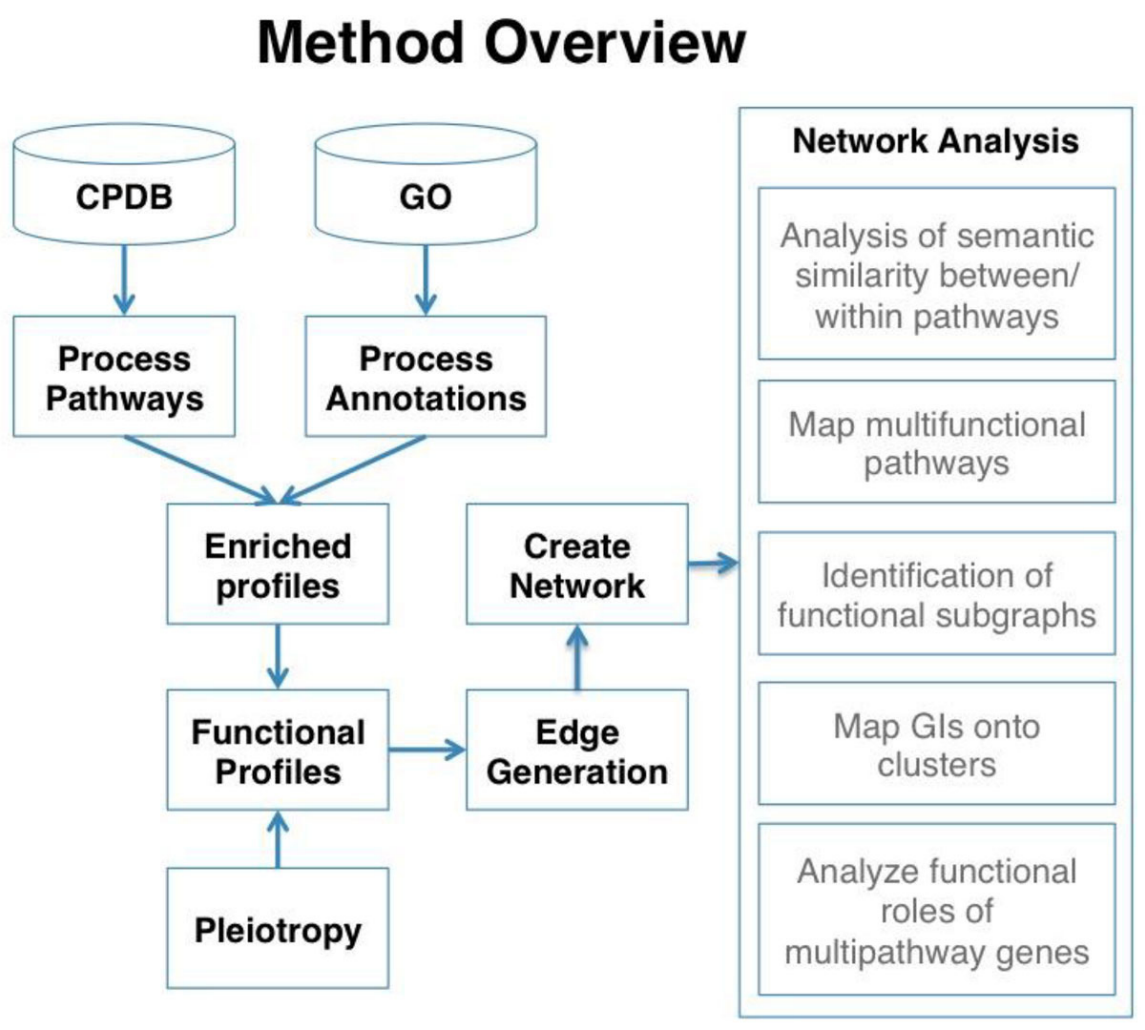
**Outline of methods used in the construction of the network and network analysis**.

Gene annotation data was integrated with pathway data to produce a set of annotated pathways, which were assembled into a functional network and analysed. An outline of the methods is given in Figure 1.

### Pathway data

*S. cerevisiae *pathway names and their constituent genes/proteins were retrieved from ConsensusPathDB (CPDB) ([17]. Pathways were represented as sets of genes. The original data set consisted of 1050 pathways with 2114 genes.

CPBD collects pathway data from multiple databases, which results in a large degree of pathway duplication and overlap, making pathway consolidation necessary [18]. Three types of data duplication were identified: duplicated pathway names, duplicated gene sets, and small pathways that were subsets of larger pathways. Databases resourced by CPDB may assign slightly different gene sets to identical pathway names, as a result of varying pathway boundaries. Repeated pathway names were identified and amalgamated into single entities by merging the gene sets. Pathways with identical gene sets were identified and redundant pathways were removed.

The gene/protein sets of some pathways were found to be subsets of larger pathways. Dealing with this form of data duplication is more complex, as the choice of which pathway to retain is not obvious. The pathways retrieved from CPDB were also highly variable in their size (see Table 1 standard deviations). To reduce this variability and ensure pathways with high functional specificity were conserved, the pathway whose size was closest to the median pathway size was retained (min (|length of pathway 1 - median|, |length of pathway 2 - median|)).

**Table 1:** Transformation of data during processing.

	Original data	Duplicated names merged	Short (<3) pathways removed	Duplicate gene sets removed	Pathway subsets removed	Unannotated genes removed
Total pathways	1050	990	715	553	272	271

Number of unique genes	2114	2114	2113	2113	1565	1433

Median genes per pathway	5	5	8	8	7	6

Mean genes per pathway	11.9	12.2	16.3	17.6	11.4	10.2

Standard deviation	23.2	23.9	27.0	28.8	16.5	13.05

Pathways containing less than three genes/proteins were considered too small for reliable statistical analysis of function and were removed. The effect that our processing had on the data set is documented in Table 1. The final data set consisted of 271 pathways and 1433 genes, with a median of six genes/proteins per pathway.

### Generation of a full set of GO identifiers for each gene

Functional gene annotations were retrieved from the Gene Ontology [19]. GO terms were assigned to the genes within each pathway. Only experimentally derived annotations or annotations generated using sequence orthologs were used, leaving 132 (9%) of genes unannotated (Table 1). Unannotated genes were omitted from the data set. To increase annotation completeness, the GO hierarchy was downloaded and parent annotations were added to genes.

### Removal of uninformative GO terms

The hierarchical nature of the Gene Ontology resulted in some annotations being too general and frequent to be considered informative. For this reason, and based on assessment of the GO annotation frequencies across the genes in the data set, annotations present in over 50% of genes were removed; these deleted annotations are listed in Additional File 1. These annotations are highly unlikely to be identified as enriched within a single pathway during later processing stages. Removing them at this point reduces repeated testing.

### Annotation of pathways

GO annotations associated with pathway genes were used to infer the function of the CPDB pathways. Only biological process annotations were used, molecular function and cellular component information were not incorporated. The Shapiro test [20] was performed to ensure that none of the GO terms were randomly distributed across the pathways (p << 0.001). Enrichment profiles were created to include all the GO terms enriched within a pathway's genes. Functional profiles were then generated to show the most specific enriched GO terms capable of describing the gene set. Functional profiles should therefore be considered as describing the main functional roles of each pathway, at the highest level of specificity possible.

### Enrichment profiles

Functional enrichment profiles were created using Fisher's exact test to identify all annotations enriched to a p-value of 0.01, within the pathway's gene set. The parameters used were: instances of the GO annotation within the pathway (how many genes the annotation was attributed to), instances of other GO terms in the pathway, instances of the annotation outside the pathway, and instances of all other GO terms outside the pathway. Using an enrichment score of 0.01 as the threshold for allocating GO terms, annotations are assigned at 99% specificity. Rather than correcting for multiple testing, we use later processing stages to remove false positive annotations, which are designed to be flexible to the varying specificity of GO term-pathway relationships. P-values gained from Fisher's exact tests are therefore referred to as enrichment scores.

### Functional profiles

The functional profile of a pathway is defined as a reduced set of enriched GO annotations that give maximum representation of a pathway's genes. Enriched annotations that were only present in one gene/protein within the pathway were excluded, as they are likely to be spurious and give a poor representation of the pathway's function.

**Figure 2:**
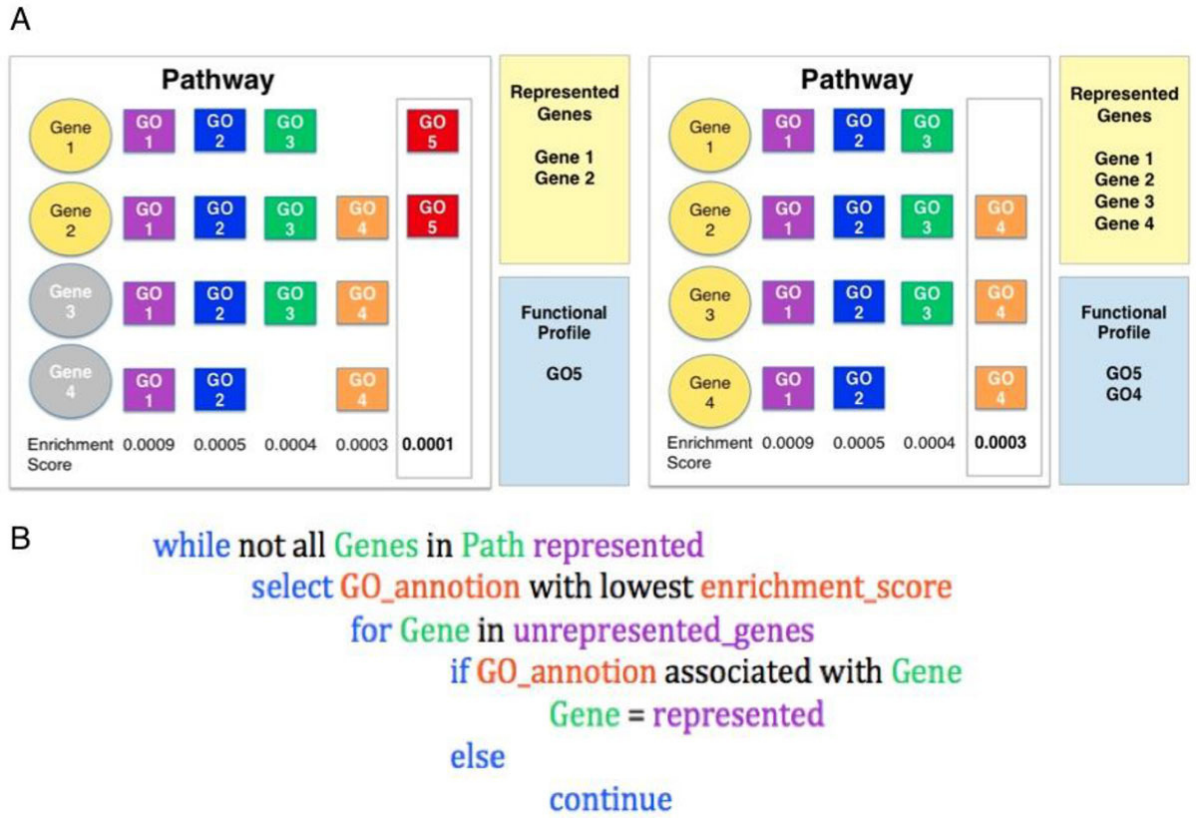
**Functional Profile creation**. (A) The figure shows one pathway, with genes represented as circles and gene annotations shown in boxes to the right of each gene. The aim of the algorithm is to select the minimum number of GO annotations necessary to represent all the genes in the pathway, preferentially selecting annotations with low enrichment scores. In this example GO 5 is the annotation with the lowest enrichment score and is therefore selected first. GO 5 is associated with genes 1 and 2, therefore GO 5 is sufficient to represent these genes. GO 4 is selected next and represents genes 2, 3, and 4; therefore GO 4 and GO 5 represent all the genes in the pathway.

The remaining annotations in each pathway's enrichment profile were considered for inclusion in the functional profile, by the ranking of their enrichment score (lowest enrichment scores first). The first GO term is selected and checked against the annotations of each gene/protein in the pathway (Figure 2). Genes associated with the annotation are considered represented. If all genes were not represented by the first GO term, GO terms associated with the remaining genes were considered. Any genes connected with this GO term were then considered represented. This process was continued until all genes were represented or until all the GO terms with significant enrichment scores were utilised. This resulted in a set of functional profiles with a median of two annotations per pathway.

### Pleiotropic genes within pathways

Pleiotropy describes genes that contribute to more than one phenotype, implying that the gene/protein is involved in more than one function. This may be due to presence of the gene/protein in different pathways, or the genes within a single pathway affecting multiple functions [21], resulting in pathway multi-functionality. These additional functions may be missed in the initial formation of functional profiles, as only the most enriched annotations for each gene set are included. A second processing stage was added to capture pleiotropic annotations. Semantic distances between GO terms were taken from Ames et al. (2013) [16]. Semantic distances were available for 88% of GO annotations within the enriched profiles. Identifying phenotypic pleiotropy is complex, as the distinction between different characters and multiple attributes of a single character is often unclear [22]. To ensure that the terms we add are truly pleiotropic we have chosen to use only terms that are semantically very different from existing terms in the functional profile.

Within functional profiles, the median semantic distance between pairs of GO terms was 6 and 95% of GO term pairs had semantic distances above 11.2. Therefore a semantic distance of 11.2 was used as the measure of pleiotropy. To avoid false positive annotations, GO terms from enriched profiles were only considered pleiotropic if they had an enrichment score below 0.0005. The semantic distance between each GO term in each pathway's enriched profile and all the GO terms in the functional profile was measured. Any enriched annotations that had a distance greater than 11.2 from all of the GO terms in the functional profile, were considered pleiotropic and added to the functional profile. Using these parameters 32 GO terms were added to 25 pathways.

**Figure 3:**
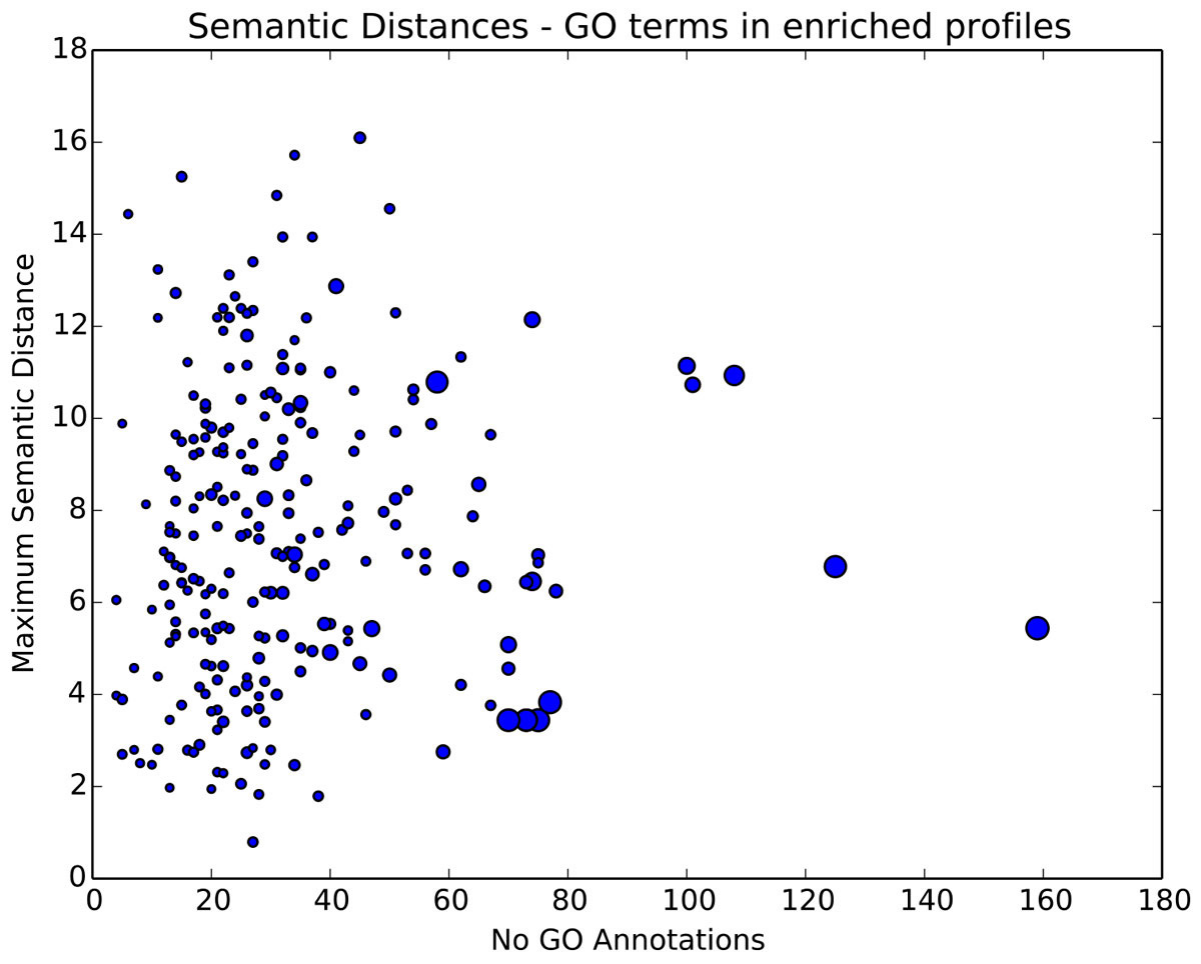
**Annotation variability within different sized pathways**. The Y-axis represents the maximum semantic distance between GO terms in each pathway's enriched profile. The X-axis represents the number of GO terms in the pathway's enriched profile. Circle size indicates the number of genes in the pathway. Pathways with low numbers of genes and annotations can be seen achieving high maximum semantic distances.

A concern when adding pleiotropic terms was that large semantic distances may be more likely to arise in larger pathways with more genes, resulting in less specific pathway functions. Plotting the number of GO annotations versus maximum semantic distances between annotations in enriched profiles (Figure 3, where circle size indicates the number of genes in a pathway), shows that although pathway size is linked to the number of enriched GO terms, it does not affect the maximum semantic distance between the terms. Terms in several small pathways' pass the threshold distance of 11.2, indicating that small pathways can contain semantically diverse enriched terms, which if omitted from the functional profile, could result in useful information being lost.

### Network generation

The annotated pathways were used as nodes and linked by shared functionality into a network. Edges were created using the Jaccard similarity coefficient to measure proportional overlap between pairs of pathway annotations (Equation 1). Jaccard coefficient scores were used to weight the edges in an undirected network.

(1) $$J\left(A,B\right)=\frac{\left|A\cap B\right|}{\left|A\cup B\right|}$$

where A and B are two sets of GO terms.

### Linking functionally similar annotations

Due to the size and hierarchical nature of GO it is possible that multiple annotations may describe very similar cellular functions. Pathways with different annotations describing highly similar functions would not be linked, therefore the network would fail to represent the pathways' functional similarity. To overcome this issue, links have been created between nodes with semantically similar annotations below a threshold (*T*) of 0.8 (Equation 2). We calculated pairwise similarity scores (*S^ab^*) between GO terms (*a *and *b*) of pairs of pathways (A and B), retaining only scores below *T*. The retained similarities were normalised, then summed to give a value (*V_AB_*) expressing the total similarity between the annotations in both pathways (if a GO annotation appeared in both functional profiles it was not compared to itself). The resulting value was then divided by the number of possible GO term pairs, to obtain the edge weight (*W_AB_*). GO term pairs with scores below the threshold of 0.8 represent the most extreme cases of semantic similarity (<0.1% of semantic distances), ensuring that the majority of the edges in the network represent identical shared annotations (74%).

(2) $$\sum_{a\in A,b\in B}\frac{T-S^{ab}}{T}\;\;W_{AB}=\frac{V_{AB}}{\left|A|*|B|-|A\cap B\right|}$$

### Genetic interaction analysis

GIs frequently occur between genes/proteins in pathways that share functions [10]. Based on this knowledge it is expected that topological clusters (see Additional File 2) in the network will be enriched for GIs. This was tested using a set of GIs from BIOGRID [23]. Excluding GIs involving genes that were absent from the data set resulted in a list of 29,309 GIs. For each GI, the set of pathways that each gene/protein participates in was retrieved, and all pathway combinations were examined. If both genes/proteins appeared in a single pathway, a within-pathway GI was recorded, whereas if each gene/protein appeared in a different pathway but the pathways were in the same cluster, a within-cluster GI was recorded. GIs linking pathways from different clusters or involving unclustered pathways were recorded as uncharacterised.

### Characterising the profiles of multi-pathway genes/proteins

To establish whether genes/proteins acting in multiple pathways are performing different roles, we performed pairwise comparisons of semantic distances between the annotations in multiple functional profiles. The sum of the semantic distances was divided by the number of genes in the profiles' union.

### Generation of the gene/protein overlap heat map

Many proteins were present in multiple pathways. To examine the relatedness of these pathways' functions, a heat map was created to compare gene/protein overlap against functional similarity. Pathways were arranged into a tree based on functional similarity, shown on both axes. This was calculated by carrying out pairwise comparisons of all GO terms between functional profiles, and taking the mean semantic distances. The tree structure was created by QuickTree using the Unweighted Pair Group Method with Arithmetic Mean joining method [24]. The heat map was created by calculating the percentage of gene/protein overlap between pathways and colouring cells accordingly.

## Results and discussion

We produced a set of functionally annotated pathways, which were assembled into a network to show functional organisation. The major functional subgraphs are identified and the relationship between functions is discussed. The functional variability of genes/proteins that participate in multiple pathways is evaluated. GI enrichment within network clusters was measured.

Biological functions require the cooperation of multiple genes and proteins. Most functional representations associated with individual genes/proteins are derived from the curation of scientific papers [25] which focus on small numbers of genes making them highly idiosyncratic and often failing to capture the cooperative aspect of biological function. In order to create systems-wide models that are more suitable to biological interpretation and understanding, new representations are needed that better reflect the cooperative nature of function. Biological pathways are a suitable candidate for higher-level representation of biological function, since they group genes and proteins that interact to produce a specific cellular or physiological outcome.

### Generation of a functionally representative set of pathways

A set of 1050 *S. cerevisiae *pathways was obtained from CPDB and processed to remove data duplication and reduce the range of pathway sizes (pathway sizes in the original data set ranged from 1 to 310). Removal of duplicated pathway names and gene sets, as well as pathways containing fewer than three genes, reduced the number of pathways in the data set to 553 (Table 1). Further processing of duplicated data selectively removed pathways whose size deviated from the median, helping to reduce the standard deviation from 23.2 in the original data set to 13.1 in the final data set. The largest pathway in the original data set was 'Metabolism' containing 310 genes, which would have dominated much of the network. The largest pathway in the final data set was 'Protein processing in endoplasmic reticulum' with a more comparable 78 genes.

### Assignment of Gene Ontology Terms to Genes

Annotations were available for 92% of genes in the data. Adding parent annotations to the GO terms initially assigned to the genes increased the median number annotations from two to 38 and the maximum from eight to 149.

Removing highly frequent, uninformative annotations from the data set reduced the median number of annotations per gene from 38 to 31. Within this final data set the range of annotations assigned to genes was large, ranging from one to 208; 75% of genes had between 14 and 66 annotations. This variability may be due to genes being attributed GO terms with large numbers of parent annotations or gene/protein multi-functionality.

### Generation of functional profiles of pathways

Fishers exact test produced large numbers of overrepresented GO terms for each pathway (median 26, range 1-159). This is in part related to the hierarchical nature of the Gene Ontology, implying that many of these annotations are describing a small number of functions at various levels of detail. Functional profiles were created to give a succinct representation of each pathway's specific functions, by selecting a reduced set of GO terms to describe the maximum number of genes/proteins inside each pathway (median 2, range 1-9). Only 35% of pathways were described by a single GO term, demonstrating that functions defined by the Gene Ontology cannot be directly mapped onto pathways, as the relationship is more complex. A moderate correlation was found between the number of genes/proteins in a pathway and the number of GO terms in its functional profile (coefficient 0.5). The majority of pathways had unique functional profiles, however 13% of functional profiles were not unique to a pathway indicating that some GO functions may be shared by discrete groups of pathways.

### Improved functional profile comprehensiveness through incorporation of gene pleiotropy

The functional profile algorithm (see Methods) selects the most enriched annotations for genes/proteins within the context of each pathway; however, multiple functions performed by genes/proteins may be missed. As a result of incorporating pleiotropic terms, 32 additional annotations were added to 25 pathways, with each pathway receiving between one and three terms. Examples of the information added by including pleiotropic terms are given in Table 2.

**Table 2:** Examples of the data added through the inclusion of pleiotropic genes.

Pathway	Original Annotations	Pleiotropic Annotations
sucrose degradation	cellular carbohydrate catabolic process	fructose import

trehalose degradation II	cellular carbohydrate catabolic process	glucose import

mannose degradation	fructose import	fructose metabolic process

The annotation overlap across the pathways illustrates the functional overlap of these pathways. Sucrose is degraded into fructose and trehalose is degraded into glucose, prior to cellular import [35]. Mannose and fructose are both transported into the cell by hexose transporters and degraded into Fructose-6-phosphate.

**Figure 4:**
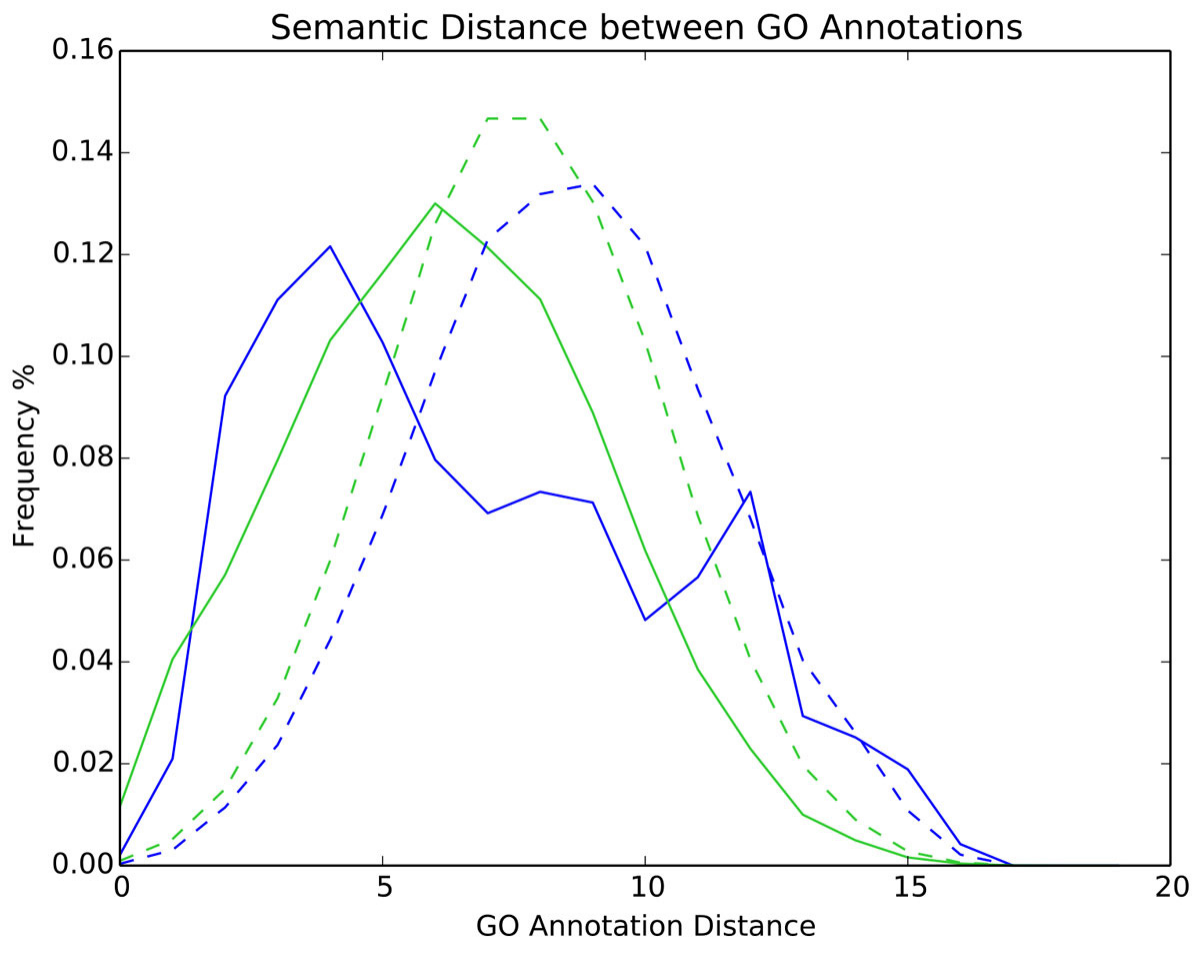
**Semantic similarity of GO annotations within/between functional and enriched profiles**. The solid blue line shows the frequency of distances between pairs of GO terms within each pathway's functional profile. The solid green line shows the frequency of distances between pairs of GO terms within each pathway's enriched profile. Annotations in functional profiles were merged and distance frequencies are shown by the dashed blue line. This process was repeated for the enriched profiles to create the dashed green line. Merging profiles gives the random expected distance between annotations, controlling for annotation frequencies. When merging profiles annotations appear multiple times, however, annotations were not compared to themselves.

We analysed the semantic distance between GO terms co-occurring within functional profiles (Figure 4). The distribution of semantic distances indicates that functional profiles have a much higher proportion of close GO terms than enriched profiles. The most frequent (mode) semantic distance between GO terms in functional profiles is four (median 6.1), which is notably lower than in enriched profiles (mode 6, median 6.2). Merging the GO terms from within functional profiles and within enriched profiles gives the distribution of semantic distances between random pairs of annotations, accounting for annotation frequency. Both functional and enriched profile sets contain many more semantically close genes/proteins than expected from chance (modes 9 and 7 respectively). Although most functional profiles contain semantically similar annotations, some are functionally diverse, as shown by the tail of the functional profile distribution (Figure 4). The spike in frequency seen at the semantic distance of 11-12 is due to the addition of pleiotropic annotations. A peak is also seen at a semantic distance of 8-9, corresponding to the mode distance in combined enriched profiles. This indicates that the pathways may incorporate a second cellular function, possibly acting as functional bridges, facilitating cellular coordination.

### Functional diversity of pathways

**Figure 5:**
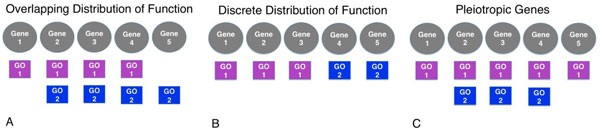
**Distribution of multiple functions across genes within pathways**. Functionality may be distributed across a pathway's genes in the following ways: pathways may have multiple functions distributed across overlapping genes (A); multiple functions may be divided into discrete (disjoint) sets of genes (B); or pleiotropic genes may have multiple layers of functionality (C).

Multiple functions can be distributed across the genes/proteins within a pathway in three ways. Functional profile annotations are either distributed across overlapping, discrete (disjoint) or pleiotropic sets of genes within the pathway (Figure 5 A, B & C respectively). The majority of pathways (84%) had all of their functional profile annotations distributed across overlapping gene/protein sets. This overlap of functions illustrates how information is passed from one function to the next, connecting cellular functions. Instances where a pathway's genes are split into discrete functional groups may indicate that the boundaries of pathway are in discord with the functional boundaries presented by the Gene Ontology. This discrete distribution of function occurs in 26 pathways, many of which are positioned in areas of the network involved with energy production and amino acid metabolism. These pathways have a median of three GO terms and the semantic distances between GO terms are higher than those observed within other pathways (median 10). Pleiotropic annotation distributions were created by the addition of pleiotropic terms following initial functional profile creation, which were present in 25 pathways.

**Figure 6:**
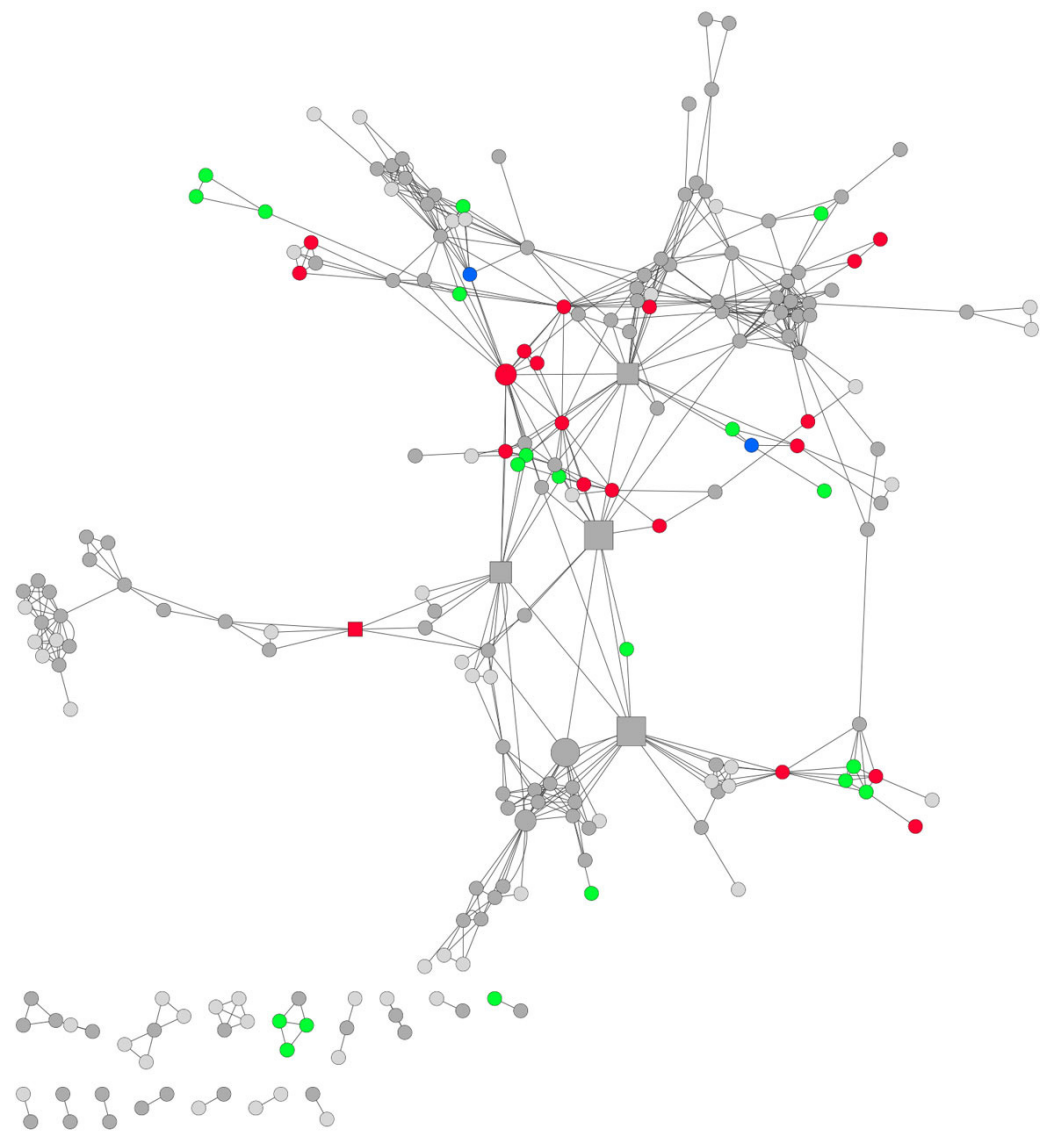
**Networks of functional links (edges) between pathways (nodes)**. Node colour indicates functional organisation of genes within the pathway: the network shows the position of pathways with overlapping functionality (dark grey), discrete functionality (red), pleiotropic genes (green) and a single function (light grey). Nodes showing discrete functionality (see Figure 5B) and pleiotropic genes (see Figure 5C) tend to form clusters and are particularly frequent within energy metabolism. Pathways with both discrete and pleiotropic function (blue) are seen linking discrete and pleiotropic pathways. The size of the nodes indicates the number of GO annotations attributed to each pathway. Square nodes indicate the five pathways with the highest betweenness centrality. Pathways with as few as two annotations have discretely distributed functionality or pleiotropic genes.

The five pathways with the highest betweenness centrality (range 0.33 to 0.19, indicated by square nodes in Figure 6) indicate pathways that are particularly important for the transfer of information within the cell. Betweeness centrality was highest in: endocytosis; glyoxylate and carboxylate metabolism; mitochondrial protein import; toll-like receptor cascades; and adenosine ribonucleotides *de novo *biosynthesis. Endocytosis is the process by which the cell imports proteins and lipids from the cell surface and links the cell membrane and signalling pathways to the metabolic pathways (Figure 6) [26]. Glyoxylate and carboxylate metabolism is necessary for the cell to grow on fatty acids and C2-compounds such as ethanol and is in centre of the network between lipid metabolism and energy metabolism (Figure 6) [27]. Mitochondria participate in several metabolic processes and import the majority of their proteins. The proteins required depend on the metabolic process taking place, therefore mitochondrial protein import connects many cellular functions and is in the centre of the network (Figure 6) [28]. Toll-like receptor cascades are essential for the cell to respond to pathogens [29]. Within the network this pathway connects cell membrane and signalling pathways to the main body of the network (Figure 6). Adenosine ribonucleotides *de novo *biosynthesis is necessary for transcription, DNA repair and replication. This pathway links gene expression to nucleotide biosynthesis.

### Functional network subgraphs

**Figure 7:**
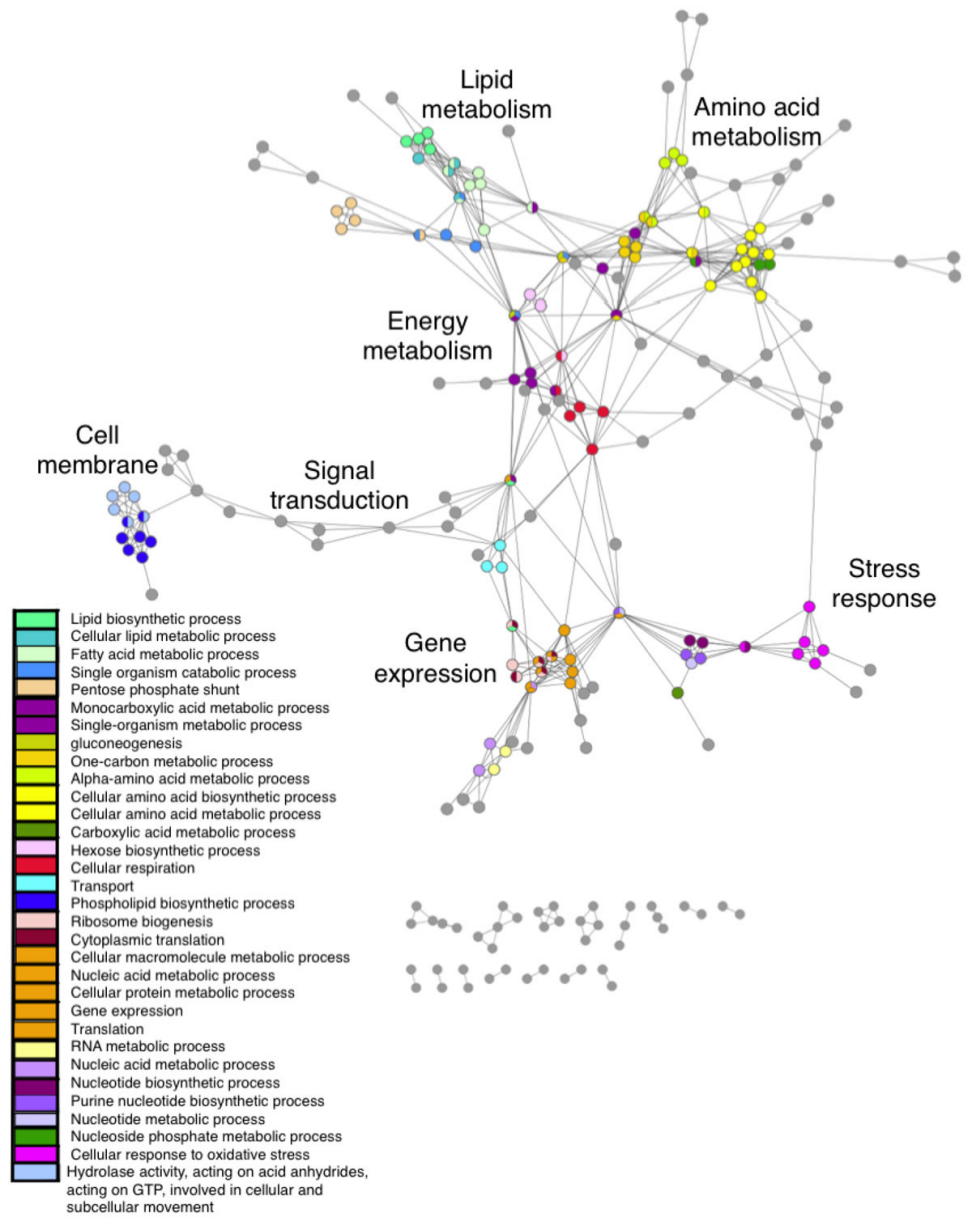
**Networks of functional links (edges) between pathways (nodes), showing major functional groups**. This figure depicts the same network as shown in figure 6, portraying the major functional groups. Colours represent frequent GO terms within the network. Pathways with less frequent GO annotations are shown in grey. GO terms linked as semantically close (equation 2) have been attributed the same colour. Labels show the major functional communities.

By mapping the most frequent GO terms onto the network of pathways, functional groups of pathways are clearly observed (Figure 7). Groups of pathways are formed involving genetic processes, metabolic processes and signalling. Energy metabolism is in the centre of the network, reflecting the necessity of energy to all biological functions (Figure 7). Transcription and nucleotide processes dominate one side of the network, with protein and lipid metabolism on the other. Cell signalling forms a detached branch attached to the main body of the network by cellular transport processes. Functional maps created by others using yeast PPI data also found that cellular communication and signal transduction were highly segregated from the rest of the network [5], while the network constructed by Yook et al. (2004) placed cellular organisation and transcription together rather than energy metabolism at the centre of the network. Protein synthesis was found to be the least connected functional module, whereas in our network protein synthesis pathways are found within the main body of the network.

A further difference between our network and PPI networks is that PPI networks tend to be hub-based networks, the network topology dominated by a small number of highly connected hub proteins and having scale-free properties [5,30,31]. Scale free distributions, characterised as having a power law degree distribution of P(k) ~ k^-γ ^where γ is typically between 2 and 3 are common in both biological and non-biological networks [32]. Within our network hub nodes would be expected to appear as highly multifunctional pathways. However, although the degree distribution did follow a power law distribution (γ = 1.3), the low gamma value indicates that none of the nodes are disproportionately influential.

### Co-occurrence with genetic interactions

**Figure 8:**
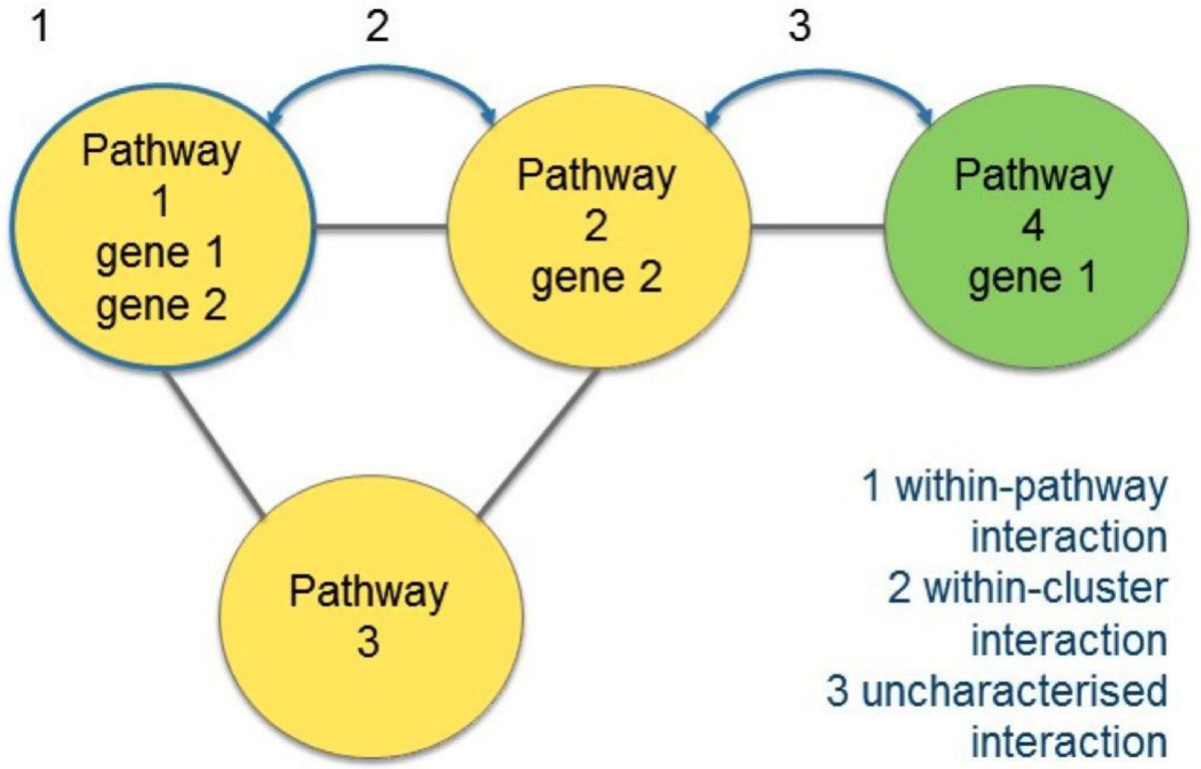
**Genetic interactions (GIs) within pathways and pathway clusters**. GIs are classified depending on whether the genes are in the same pathway, within two pathways in the same network cluster, or uncharacterised (between two pathways in different clusters or involving unclustered pathways). Genes present in multiple pathways will result in GIs appearing in many pathway pairs. In these situations all pathway pairs are classified separately. The yellow nodes show three pathways in a single cluster. The green node represents a pathway in a separate cluster. All possible ways of connecting gene 1 and gene 2 across all pathways are explored.

GIs tend to occur within pathways and between functionally similar genes [8,10]. It is therefore expected that pathway-clusters within the network will be enriched for GIs. To test this the proportion of GIs that occurred within pathways and within clusters (Figure 8) was compared to output from randomised GI data (Table 3). We find GIs within pathways were increased by a factor of 6.5 compared to randomised data and within-cluster GIs were enriched by a factor of 5.5. The topological network clusters are shown in Additional File 2. This confirms the biological significance of the network's organisation.

**Table 3:** Enrichment of GIs within pathways and network clusters.

	Genetic interaction data	Randomised data
Within-pathway	5.45%	0.840%

Within-cluster	4.37%	0.800%

Percentages of within-pathway or within-cluster GIs, compared to randomised interactions.

### Pathway dependent gene/protein multi-functionality

**Figure 9:**
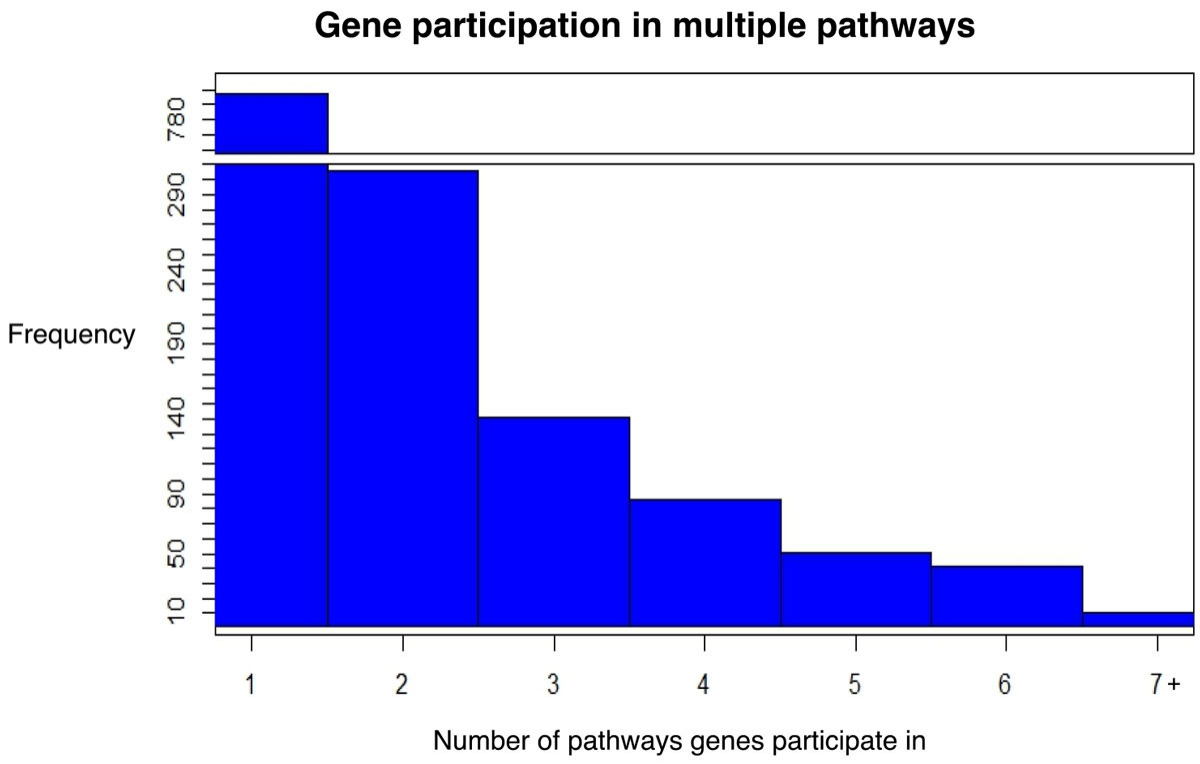
**Frequency of gene participation in multiple pathways**. Of the 1433 genes in the network, 797 (56%) genes were found in one pathway, 304 (21%) of genes were found in 2 pathways, 332(23%) genes were found in 3 or more pathways.

**Figure 10:**
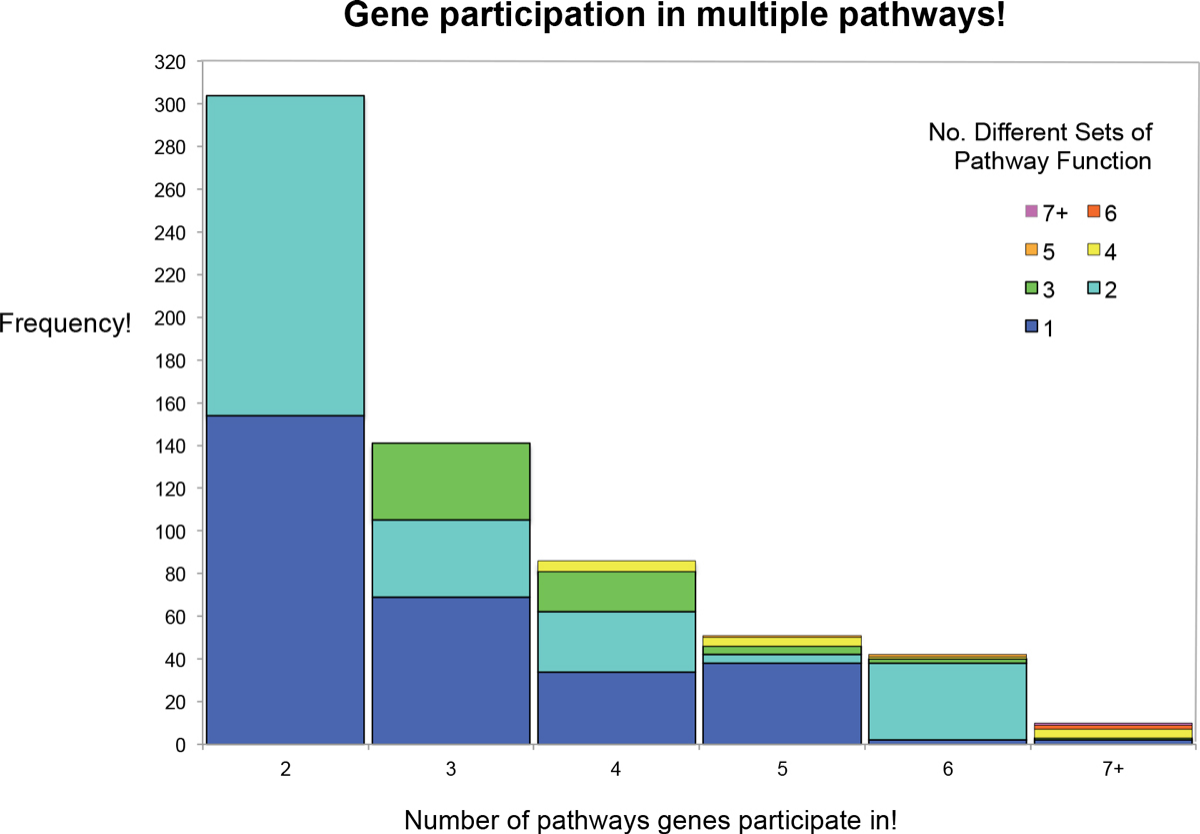
**Functional variability of multi-pathway genes**. Bars indicate the number of pathways that multi-pathway genes participate in. Bar colours indicate the number of discrete functional profiles (no GO terms overlap) associated with genes' pathways

Of the 1433 genes/proteins in the data set, 44% were found in multiple pathways *(*Figure 9), with the maximum number of pathways a gene/protein appeared in being 11 (gene AAT2). If genes/proteins perform different functions in the context of different pathways then the functional profiles of these pathways will be different. Within our data set, 83% of multi-pathway genes have distinct functional profiles for each pathway they participate in. Pathway profiles were considered distinct if they did not contain identical sets of GO terms; however overlapping annotations were allowed. Annotation overlap between functional profiles is expected to be partially due to physical overlap between the pathways. Figure 10 shows the number of discrete (disjoint) sets of functional annotations found in genes participating in multiple pathways. Two or more discrete gene sets are frequently observed indicting that the genes are participating in distinct, context dependent pathways.

**Figure 11:**
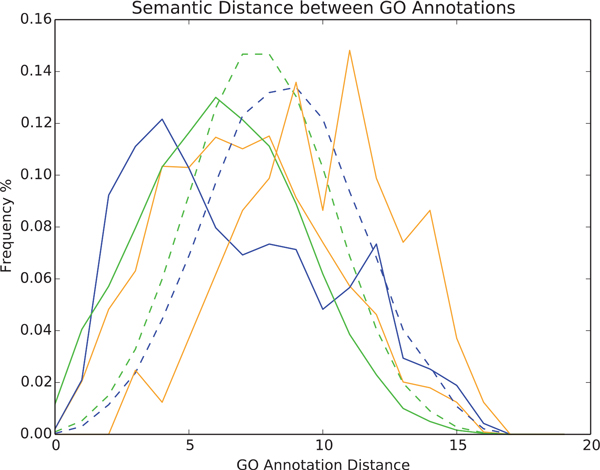
**Semantic distance between multi-pathway genes' functional profiles**. The solid blue and green lines show the frequency of distances between pairs of GO terms, within each of the pathway profiles. The dashed lines show the frequency of semantic distances between random annotation pairs. The orange line shows pairwise GO annotation distances between functional profiles of pathways sharing a multi-pathway gene. Comparison to the solid blue line shows increased semantic distances between the functional profiles of multi-pathway genes.

To further explore the possibility that genes/proteins acting in different pathways have different functions, we calculated semantic distances between the functional profiles of multi-pathway genes/proteins (Figure 11 orange line). The mode distance between functional profiles is 4 showing that many of these pathways have highly similar profiles. These are likely to represent overlapping pathways. However, semantically distant GO terms (scoring between 5 and 11) were much more common between functional profiles than within functional profiles (blue line). This indicates that the pathway dependent functions of multi-pathway genes/proteins are frequently very different and the peak in frequency at a semantic distance of 8 indicates that these pathways may be as functionally unrelated as two pathways selected at chance.

**Figure 12:**
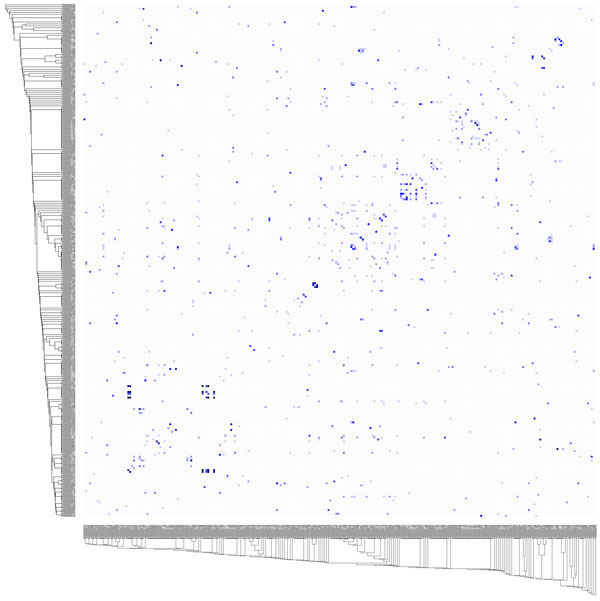
**Relationship between pathway functionality and gene/protein overlap**. Trees show pathways clustered by functional similarity. The heat map shows gene/protein overlap. Dark cells from the diagonal showing pathway self-comparison have been removed. Dark cells along diagonal show that similar pathways are likely to share genes, showing some degree of pathway overlap in the data set. Dark cells positioned away from the diagonal show functionally unrelated pathways sharing genes.

Finally we examined the relationship between gene/protein overlap and pathway function by organizing pathways based on functionality then considering gene/protein overlap (Figure 12). Functionally divergent pathways can be seen sharing genes, indicating that some genes perform different roles depending on pathway context.

### Comparison to Over Representation Analysis

To validate our results we compared them to DAVID [33], a tool commonly used for over representation analysis (ORA). We used DAVID to group genes based on GO annotation similarity. We then measured the number of shared annotations between genes from the same or different ORA groups. Gene pairs within the same ORA groups shared a mean of 3.9 annotations (n 63143) while genes in different ORA groups shared a mean of 0.6 (n 764398), indicating that the edges within our network are strongly supported by DAVID functional groupings (Welch's T-test gave p = 0.0).

### Limitations

Our method produces pathway annotations from GO data and organises pathways into a network representation of cellular function. The network contains 271 pathways, covering a wide range of functions including metabolism, signal transduction, gene expression and DNA maintenance. Yeast has 6604 genes of which 5151 are characterised [34], therefore the 1433 annotated genes analysed within the pathways of this network should not be considered as complete coverage. Our method can however be adjusted to allow more genes and pathways into the final data set, or to study specific sets of pathways. The highly frequent GO terms in Additional File 1 highlights the bias towards metabolic pathways within the data set.

## Conclusion

We have developed a method for organising cellular processes based on function, which accounts for temporal interactions modelled through pathways and allows multifunctional genes to be portrayed independently in their different biological contexts. The network illustrates the organisation of function, as multiple pathways co-operate to ensure cellular processes are coordinated. Pathway multi-functionality was examined, determining that pathways vary greatly in the number and diversity of GO functions they facilitate. The functional variability of genes within multiple pathways was also demonstrated. Appreciation of multi-functionality at the level of both genes and pathways is critical for understanding pleiotropic genes and their relationship to multiple phenotypes, interpreting GIs and considering the transfer of information within the cell. Our representation of cellular function will enable analysis of gene/protein activity in the context of their functional roles, instead of the typical molecule-centric approach. This method can be adapted to incorporate different data types into the network, such as expression data and genetic interaction data. Future work will include incorporation of expression data to create directed edges showing the information flow between edges.

## Competing interests

The authors declare that they have no competing interests.

## Authors' contributions

Conceived and designed the project: DR, GN and JMS. Provided semantic distance data: RMA. Constructed the network: RAS, with input from DR, GN, JMS. Pathway and network analysis: RAS and RMA. Wrote the paper: RAS, with input from DR, GN, JMS and RMA.

## Supplementary Material

Additional file 1 **Table S1: GO annotations considered too frequent to be informative (>50% of annotations) and removed from the data set**. Click here for file

Additional file 2 Figure S1: Network clusters created using ONECLUST. The Cytoscape plugin ClusterONE was used to calculate network clusters, using weighted edges and a minimum cluster density of 0.25 to include all the main clusters. (*.pdf). Click here for file

## Declarations

The publications charges for this article were funded by an RCUK block grant to the University of Manchester.

This article has been published as part of *BMC Systems Biology *Volume 9 Supplement 6, 2015: Joint 26th Genome Informatics Workshop and 14th International Conference on Bioinformatics: Systems biology. The full contents of the supplement are available online at http://www.biomedcentral.com/bmcsystbiol/supplements/9/S6.
