# Supplementary material for: Disentangling the multigenic and pleiotropic nature of molecular function
Source: BMC Syst Biol. 2015 Dec 9;9(Suppl 6):S3. doi: 10.1186/1752-0509-9-S6-S3 (PMC4674882; doi:10.1186/1752-0509-9-S6-S3)
Supplement: Additional file 1 — Table S1: GO annotations considered too frequent to be informative (>50% of annotations) and removed from the data set. [file 1752-0509-9-S6-S3-S1.pdf]

Additional file 1: Table S1: GO annotations considered too frequent to be informative (>50% of annotations) and removed from the data set.

**GO term**	**% Genes**
Biological process	100
cellular process	93
metabolic process	80
cellular metabolic process	77
primary metabolic process	77
organic substance metabolic process	76
single organism process	73
single-organism cellular process	66
