# Supplementary material for: Disentangling the multigenic and pleiotropic nature of molecular function
Source: BMC Syst Biol. 2015 Dec 9;9(Suppl 6):S3. doi: 10.1186/1752-0509-9-S6-S3 (PMC4674882; doi:10.1186/1752-0509-9-S6-S3)
Supplement: Additional file 2 — Figure S1: Network clusters created using ONECLUST. The Cytoscape plugin ClusterONE was used to calculate network clusters, using weighted edges and a minimum cluster density of 0.25 to include all the main clusters. (*.pdf). [file 1752-0509-9-S6-S3-S2.pdf]

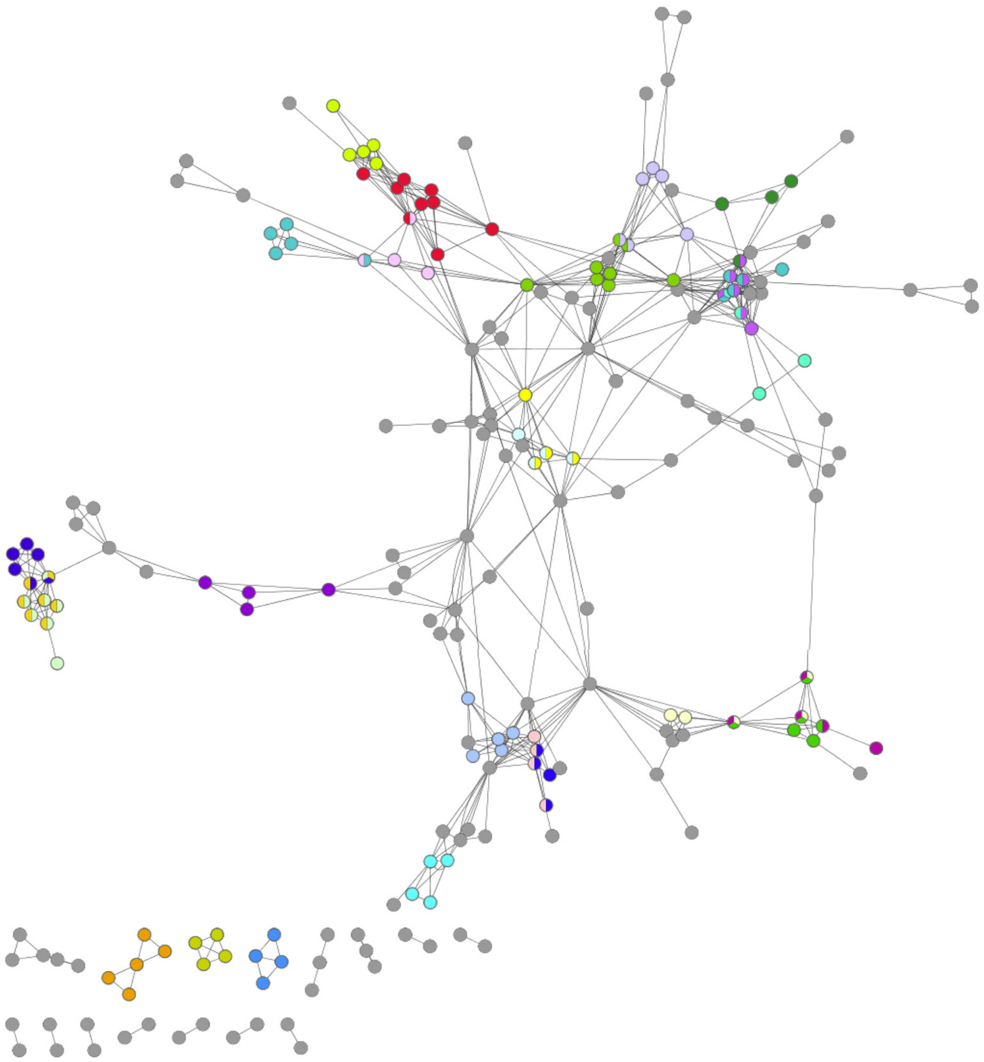

Additional File 2: Figure S1: Network clusters created using ONECLUST. The Cytoscape plugin ClusterONE was used to calculate network clusters, using weighted edges and a minimum cluster density of 0.25 to include all the main clusters.
